# Resveratrol-driven macrophage polarization: unveiling mechanisms and therapeutic potential

**DOI:** 10.3389/fphar.2024.1516609

**Published:** 2025-01-13

**Authors:** Panting Wang, Zixi Li, Yixuan Song, Bowei Zhang, Chaofeng Fan

**Affiliations:** ^1^ Department of Neurosurgery West China Hospital, Sichuan University, Chengdu, China; ^2^ West China School of Nursing Sichuan University, Chengdu, China; ^3^ School of Pharmacy, Chengdu University of Traditional Chinese Medicine, Chengdu, China; ^4^ Southwest Institute of Technical Physics, Chengdu, China

**Keywords:** resveratrol, macrophage polarization, M1/M2 phenotypes, cancer, inflammation

## Abstract

Resveratrol, a polyphenolic compound known for its diverse biological activities, has demonstrated multiple pharmacological effects, including anti-inflammatory, anti-aging, anti-diabetic, anti-cancer, and cardiovascular protective properties. Recent studies suggest that these effects are partly mediated through the regulation of macrophage polarization, wherein macrophages differentiate into pro-inflammatory M1 or anti-inflammatory M2 phenotypes. Our review highlights how resveratrol modulates macrophage polarization through various signaling pathways to achieve therapeutic effects. For example, resveratrol can activate the senescence-associated secretory phenotype (SASP) pathway and inhibit the signal transducer and activator of transcription (STAT3) and sphingosine-1-phosphate (S1P)-YAP signaling axes, promoting M1 polarization or suppressing M2 polarization, thereby inhibiting tumor growth. Conversely, it can promote M2 polarization or suppress M1 polarization by inhibiting the NF-κB signaling pathway or activating the PI3K/Akt and AMP-activated protein kinase (AMPK) pathways, thus alleviating inflammatory responses. Notably, the effect of resveratrol on macrophage polarization is concentration-dependent; moderate concentrations tend to promote M1 polarization, while higher concentrations may favor M2 polarization. This concentration dependence offers new perspectives for clinical treatment but also underscores the necessity for precise dosage control when using resveratrol. In summary, resveratrol exhibits significant potential in regulating macrophage polarization and treating related diseases.

## Graphical Abstract

**Figure:**
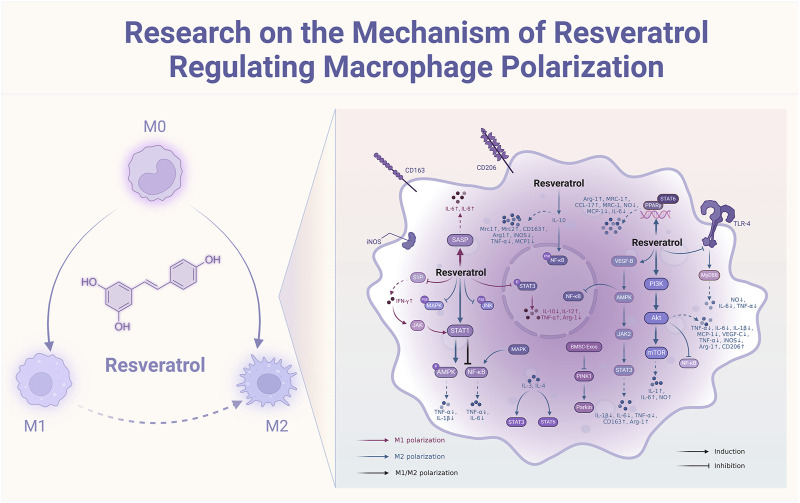
This review explores the therapeutic potential of resveratrol in disease management by assessing its ability to modulate macrophage polarization towards the M1 and M2 phenotypes.

## 1 Introduction

Resveratrol (C₁₄H₁₂O₃) is a distinguished non-flavonoid polyphenolic phytoalexin (Galiniak et al., 2019) that functions as a natural defense mechanism in plants. This compound is prevalent in various dietary sources, including grapes, peanuts, blueberries, lingonberries, cranberries, and purple grape juice (Tian and Liu, 2020; Ispiryan et al., 2024). Notably, red wine contains an average of approximately 1.9 ± 1.7 mg/L of resveratrol, with certain batches exhibiting higher concentrations. In contrast, white and rosé wines possess significantly lower resveratrol levels, ranging from 0 to 1.089 mg/L and around 0.29 mg/L, respectively (Weiskirchen and Weiskirchen, 2016), with the summary provided by Tian and Liu (2020). Extensive research has consistently elucidated that resveratrol exhibits a myriad of therapeutic properties, encompassing anti-inflammatory (Huang et al., 2024; Vestergaard and Ingmer, 2019), anti-aging (Shahcheraghi et al., 2023; Sharifi-Rad et al., 2022), anti-diabetic (Youjun et al., 2023), anti-cancer (Lafta et al., 2023; Zhang et al., 2023), and cardiovascular protective effects (Brown et al., 2024; Zivarpour et al., 2022). These compelling findings underscore the considerable potential of resveratrol as a pivotal agent in promoting health and mitigating a spectrum of diseases.

Macrophage polarization is a dynamic and crucial process that occurs when macrophages are activated by a variety of stimuli (Zhou et al., 2022), such as pathogens, inflammatory signals, cytokines, or specific physicochemical factors. This activation results in the differentiation of macrophages into two distinct subtypes: the pro-inflammatory M1 macrophages and the anti-inflammatory M2 macrophages (Kerneur et al., 2022; Mantovani et al., 2022). These specialized phenotypes play essential roles in orchestrating effective bactericidal and anti-tumor responses, as well as in modulating the initiation and resolution of diseases through intricate signal transduction pathways (Li Y. et al., 2024; Wang et al., 2021; Wang S. et al., 2024). The delicate balance between M1 and M2 macrophage polarization is vital for the maintenance of immune homeostasis. In the context of acute inflammation, macrophages differentiate into the M1 subtype, which is characterized by a robust production of cytokines that are instrumental in the elimination of invading pathogens. Once the pathogens have been cleared, macrophages undergo a transition to the M2 subtype (Chang et al., 2020). This shift is marked by the secretion of anti-inflammatory cytokines, which serve to dampen inflammation, facilitate tissue repair, and restore tissue integrity (Chen S. et al., 2023; Wynn et al., 2013). The intricate mechanism between M1 and M2 polarization is essential for the proper resolution of inflammatory responses and the maintenance of overall health.

**FIGURE 1:**
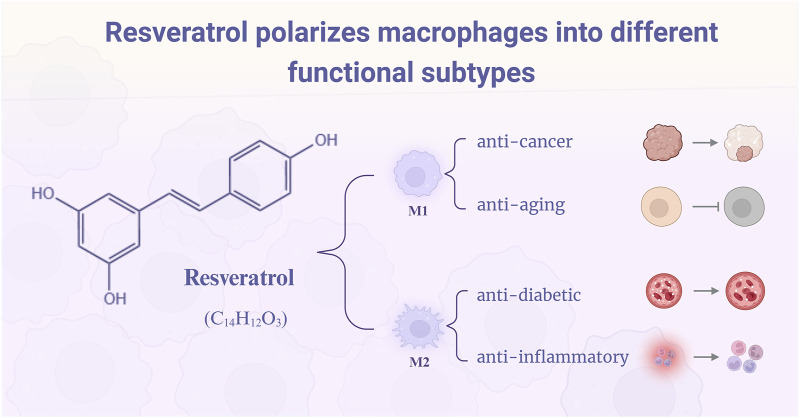
Resveratrol polarizes macrophages into M1 and M2 phenotypes with distinct therapeutic functions. By modulating macrophage polarization, resveratrol exerts its pharmacological effects: M1-polarized macrophages demonstrate anticancer and anti-aging activities, while M2-polarized macrophages exhibit anti-diabetic and anti-inflammatory actions.

**FIGURE 2:**
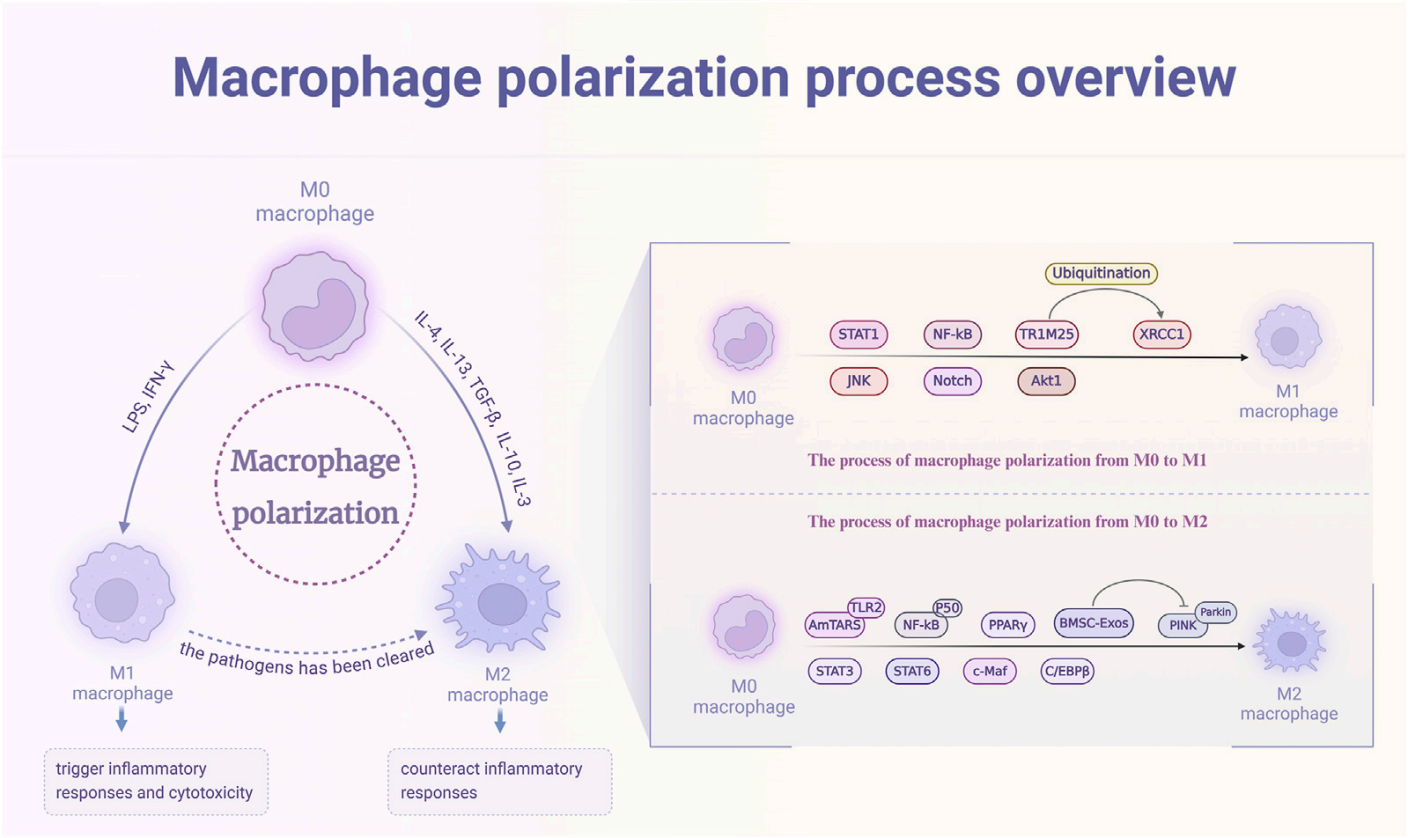
The polarization process of macrophages and its triggering targets. M0 macrophages, upon stimulation with factors such as LPS and IFN-γ, activate signaling pathways including STAT1, NF-κB, and JNK, leading to polarization into the M1 phenotype. This polarization induces inflammatory responses and cytotoxicity. Conversely, exposure of M0 macrophages to cytokines like IL-4, IL-13, and TGF-β activates pathways such as AmTAR, STAT3, and NF-κB, promoting polarization into the M2 phenotype to mitigate inflammation. Additionally, M1 macrophages can transition to the M2 phenotype following the clearance of pathogens.

In recent years, significant strides in understanding the mechanisms of macrophage polarization have brought resveratrol to the forefront as a promising clinical intervention for diseases such as inflammation and cancer (Chen and Musa, 2021; Ren et al., 2021). Emerging research suggests that resveratrol is capable of modulating macrophage polarization via multiple pathways, which could be instrumental in managing conditions like inflammation, diabetes, and cancer (Malaguarnera, 2019). A review of the literature indicates a growing interest in the study of resveratrol’s effects on macrophage polarization, highlighting the importance of its regulatory role on the M1/M2 balance as a subject of intensive scientific investigation. Despite the considerable progress made in elucidating the interplay between resveratrol, macrophage polarization, and the pathogenesis of various diseases, comprehensive overviews that integrate these findings are still relatively rare. This article endeavors to bridge this gap by summarizing the mechanisms through which resveratrol regulates macrophage polarization (Figures 1, 2). By doing so, it aims to provide novel therapeutic insights for diseases where macrophage polarization plays a critical role, potentially paving the way for more effective treatment strategies in the future.

## 2 Research progress on the mechanism of macrophage polarization in disease treatment

### 2.1 The role of macrophage M1 polarization in inflammatory responses and cytotoxicity

Studies have demonstrated that by modulating signaling pathways such as NF-κB, STAT1, c-Jun N-terminal kinase (JNK), and IFN-γ/JAK/STAT1, the polarization of macrophages into the M1 phenotype is promoted or the M2 polarization is inhibited, thereby triggering inflammatory responses and cytotoxicity.

The polarization of macrophages towards the M1 phenotype is a critical process in the immune response, and it is heavily influenced by the activation of key signaling pathways. The NF-κB and STAT1 pathways are particularly important in this context, as their activation is crucial for driving the M1 polarization. This process enhances the macrophages’ cytotoxic effects and pro-inflammatory capabilities, which, while essential for pathogen elimination, can also result in tissue damage if not properly regulated (Li et al., 2023; Mussbacher et al., 2023; Wang et al., 2014). Interestingly, the inhibition of the JNK signaling pathway has been observed to initiate M1 macrophage polarization, highlighting the complex interplay between signaling pathways in macrophage biology. Additionally, the activation of the Notch and Akt1 pathways has a dual role in macrophage polarization by promoting the M1 phenotype while concurrently suppressing the M2 phenotype, which is typically associated with anti-inflammatory and reparative functions. Furthermore, IFN-γ, an endogenous activating factor, plays a significant role in M1 macrophage polarization by activating STAT1 through the IFN-γ/JAK/STAT1 signaling pathway (Cheng et al., 2019; Li et al., 2021). This activation is particularly effective in inducing the M1 phenotype, further underscoring the importance of IFN-γ in the pro-inflammatory activities of M1 macrophages. These insights into the signaling pathways that regulate macrophage polarization provide a deeper understanding of the mechanisms underlying inflammation and tissue damage, offering potential targets for therapeutic intervention in diseases characterized by inappropriate macrophage activation.

Recent advancements in research have revealed that the targeting of TRIM25 has been shown to play a role in modulating macrophage polarization. Specifically, TRIM25 catalyzes the ubiquitination of XRCC1, which in turn promotes M1 macrophage polarization and induces programmed cell death (Blagov et al., 2023; Jing et al., 2023; Wu et al., 2024). This process is known to exacerbate atherosclerosis, further emphasizing the importance of understanding the intricate mechanisms behind macrophage polarization. These groundbreaking findings not only expand our understanding of the role macrophages play in immune responses and disease progression but also hold the potential to inform the creation of new therapeutic strategies. By targeting key regulatory genes and proteins involved in macrophage polarization, it may be possible to develop treatments that specifically address cytotoxicity-related diseases, offering hope for more effective clinical interventions in the future.

### 2.2 Research on the molecular mechanisms of macrophage M2 polarization in the treatment of inflammation

In contrast to M1 polarization, macrophage M2 polarization is regulated by signaling pathways such as NF-κB, MAPK, PI3K/AKT, STAT3, STAT6, peroxisome proliferator-activated receptor gamma (PPARγ), p50 NF-κB, and C/EBPβ, which collectively promote M2 polarization and aid in the treatment of inflammation.

The synergistic interaction between AmTARS and toll-like receptors 2 (TLR2) has been demonstrated to effectively activate the MAPK and PI3K/AKT signaling pathways. This activation leads to a significant increase in interleukin 10 (IL-10) production, which is accompanied by the inhibition of the pivotal inflammatory mediator NF-κB. Consequently, this biological response results in the amelioration of pathological manifestations in mouse models of colitis (Kim et al., 2023). Furthermore, IL-4 and IL-13 are identified as key cytokines that foster M2 macrophage polarization by engaging the STAT3 and STAT6 signaling pathways. These pathways are essential for the establishment of immune tolerance and the initiation of tissue repair mechanisms. Building upon this, IL-10 is recognized for its role in advancing M2 polarization by enhancing the activity of p50 NF-κB homodimers, c-Maf, and STAT3 (Wang et al., 2014; Xia et al., 2023). PPARγ, a lipid-activated transcription factor in macrophages, emerges as a significant player, with its dual function in lipid metabolism and the modulation of inflammatory responses. The collaborative interaction between STAT6 and PPARγ facilitates DNA binding, which subsequently regulates the expression of genes that are integral to the formation of M2 macrophage markers. Adding to this intricate regulatory network, interferon regulatory factor 3 (IRF-3) and IRF-4 are highlighted for their influential roles in M2 polarization. CCAAT/enhancer binding protein beta (C/EBPβ), a component of the C/EBP family, is noted for its ability to stimulate macrophage activation and the expression of genes specific to the M2 phenotype (Cheng et al., 2019). These discoveries provide a comprehensive view of the complex signaling events that underpin M2 macrophage polarization and offer promising avenues for therapeutic strategies aimed at modulating macrophage function in various disease contexts.

Recent studies have shed light on the role of Bone Marrow Stromal Cell-derived Exosomes (BMSC-Exos) in modulating the polarization of synovial macrophages. By inhibiting the PTEN induced putative kinase 1 (PINK1)/Parkin signaling pathway, BMSC-Exos have been shown to suppress the M1 polarization and promote the M2 polarization in the synovium. This regulatory mechanism leads to a reduction in cartilage damage in osteoarthritis rats. Concurrently, there is a decrease in the serum expression levels of pro-inflammatory cytokines such as IL-6, IL-1β, and tumor necrosis factor-alpha (TNF-α), while the level of the anti-inflammatory cytokine IL-10 increases (Li B. et al., 2024). These findings underscore the therapeutic potential of macrophage polarization in disease treatment and highlight the importance of precise regulation of macrophage polarization in managing inflammatory diseases. The ability to modulate macrophage polarization presents a promising avenue for developing novel therapeutic strategies that could revolutionize the treatment of various inflammatory conditions.

## 3 Research on the pharmacological action of resveratrol in regulating macrophage polarization for the treatment of tumors and inflammatory diseases

**FIGURE 3:**
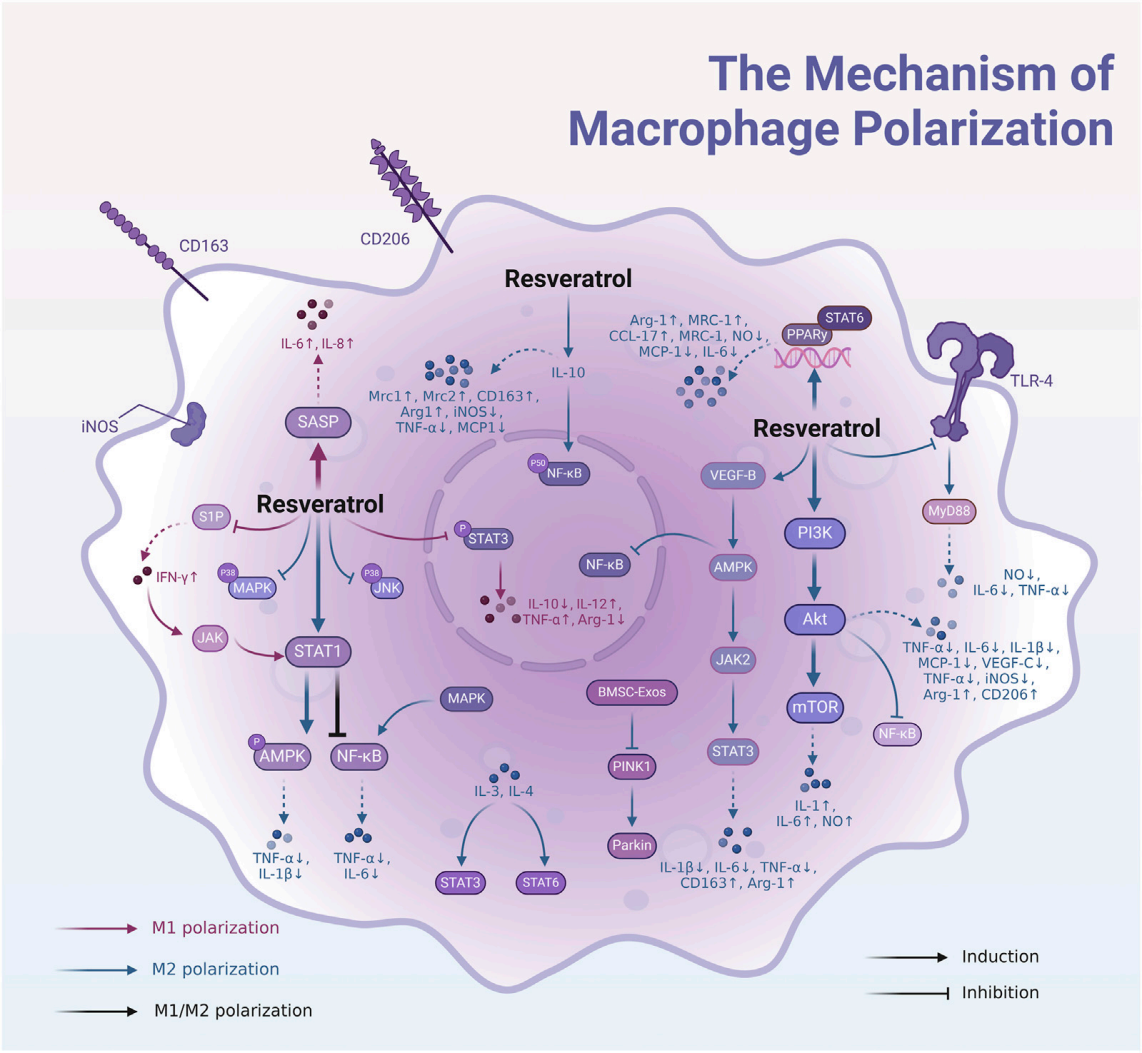
Mechanisms by which resveratrol regulates macrophage polarization through multiple signaling pathways. Resveratrol can regulate the M1 or M2 polarization of macrophages individually, or it can promote M1 polarization while inhibiting M2, or conversely, promote M2 polarization while inhibiting M1. Resveratrol promotes M1 polarization by activating the SASP and inhibiting S1P and phosphorylated STAT3. Conversely, resveratrol induces M2 polarization by activating the PI3K/Akt/mTOR pathway, enhancing VEGF expression, and stimulating AMPK to suppress the NF-κB pathway. Additionally, AMPK activation leads to JAK2-mediated upregulation of STAT3, while resveratrol inhibits TLR-4 to suppress MyD88, further promoting M2 polarization. Resveratrol also activates STAT1 to either stimulate AMPK or inhibit the NF-κB pathway, thereby reinforcing M2 macrophage polarization.

After examining the diverse mechanisms of macrophage polarization into M1 and M2 phenotypes and their roles in diseases such as inflammation and tumors, we turn our attention to how resveratrol influences these polarization processes to exert its pharmacological effects (Supplementary Table S1; Figure 3). Resveratrol has garnered extensive research interest for its ability to modulate macrophage polarization, particularly in the context of tumor and inflammatory disease treatment.

### 3.1 Resveratrol’s anti-tumor mechanism: the synergistic effect of promoting macrophage M1 polarization or inhibiting M2 polarization

The SASP is a hallmark of cellular senescence, whereas STAT3 is a transcription factor pivotal in signal transduction (Li et al., 2023; Wang H. et al., 2024). Resveratrol can modulate macrophage polarization towards the M1 phenotype by activating the SASP pathway or by inhibiting the STAT3 and S1P-YAP signaling axis, thereby suppressing cancer cell growth and proliferation.

Sun and colleagues have uncovered the intricate effects of resveratrol on macrophage polarization, revealing its potential in cancer therapy. Their research indicates that resveratrol modulates cytokine levels, with a decrease in IL-10 and an increase in IL-12 and TNF-α. Notably, a concentration of 20 μM resveratrol was found to downregulate the expression of M2 markers such as MRC1, CCL24, Chi3l3, and Retnla, highlighting its influence on M2 polarization. *In vivo* studies have corroborated these findings, demonstrating that resveratrol significantly inhibits tumor growth, a response that is associated with reduced p-STAT3 expression in tumor tissues (Sun et al., 2017; Zhang and Sioud, 2023). This suggests that resveratrol’s impact on macrophage polarization may be linked to the inhibition of STAT3 activation, a key regulator of immune responses. Further research has shown that resveratrol promotes M1 macrophage polarization by diminishing IL-6 production and suppressing STAT3 activation. It also hinders M2 macrophage polarization and impairs the differentiation of Tohoku Hospital Pediatrics-1 (THP-1) cells into the M2 phenotype, as evidenced by reduced arginase-1 (ARG1) expression, thereby limiting the proliferation of breast cancer cells (Cheuk et al., 2022). Resveratrol’s ability to modulate THP-1 cell polarization is further exemplified by its activation of the SASP pathway, leading to increased expression and secretion of IL-6 and IL-8, and inducing M1 macrophage polarization that results in senescence in clear cell renal cell carcinoma cells (Chen Z. et al., 2023).

In addition to the transformation of macrophages from M0 to M1, resveratrol can also regulate the shift of macrophages from M2 to M1. In the context of lymphoma treatment, resveratrol administration in an obesity-lymphoma mouse model has been shown to increase the F4/80+MHCII + cell subset, associated with M1 macrophages, and decrease the F4/80+CD206+ cell subset, associated with M2 macrophages. This indicates a shift in macrophage phenotype from M2 to M1, which is accompanied by downregulation of aberrant sphingosine kinase 1 (SPHK1), phosphorylated YAP, and the YAP target gene CTGF in obesity-lymphoma mice (Wang et al., 2023). These findings underscore resveratrol’s role in combating cancer by targeting the S1P-YAP signaling axis and influencing macrophage polarization. By promoting the M1 phenotype and inducing cell senescence, resveratrol exerts immune-modulating effects and demonstrates significant antitumor efficacy, positioning it as a promising agent in cancer therapy.

In a short, resveratrol modulates macrophage polarization by promoting the M1 phenotype and inhibiting the M2 phenotype through the activation of the senescence-associated secretory phenotype (SASP) signaling pathway and suppression of the STAT3 and S1P-YAP pathways. These mechanisms confer significant immunomodulatory and senescence-inducing effects, thereby exerting antitumor activity and highlighting resveratrol’s potential as a cancer therapeutic agent. Numerous clinical trials have substantiated the efficacy of resveratrol in oncology, demonstrating its applicability in the prevention and treatment of various cancer types. Owing to its exceptionally low toxicity and ability to target mutated molecules and key signaling pathways across multiple tumors, resveratrol emerges as an ideal anticancer compound. Furthermore, it may exhibit synergistic effects when combined with diverse chemotherapeutic agents and targeted therapies, enhancing antitumor efficacy (see Section 4) (Ko et al., 2017).

### 3.2 Resveratrol’s anti-inflammatory mechanism: the dual role of inhibiting M1 macrophage polarization or promoting M2 polarization

In contrast to its effects on the M1 phenotype, resveratrol regulates macrophage polarization towards the M2 phenotype through pathways such as NF-κB and AMPK/PI3K/Akt, upregulating anti-inflammatory factors and downregulating pro-inflammatory factors. This regulation helps combat inflammation, diabetes, and cardiovascular and cerebrovascular diseases.

#### 3.2.1 NF-κB pathway: a pivotal node in resveratrol-induced M2 polarization or inhibited M1 polarization

The transcription factor NF-κB is a master regulator in the activation of the inflammasome and the modulation of inflammatory responses (Mussbacher et al., 2023). Within the context of macrophage biology, the specific activation of NF-κB is a critical determinant of their polarization state (Songkiatisak et al., 2022). Resveratrol has been shown to modulate macrophage polarization by inhibiting NF-κB activation, thereby exerting its anti-inflammatory effects.

Macrophage polarization is increasingly recognized for its beneficial impact on blood sugar regulation. Resveratrol aids in the polarization of macrophages towards the M2 phenotype by influencing the NF-κB signaling pathway, which in turn inhibits inflammation. In scenarios of insulin resistance and obesity, there is an increase in the proportion of M1 macrophages, which exacerbates inflammation. Recent studies have indicated that resveratrol possesses therapeutic effects on inflammation caused by insulin resistance. Gao et al. (2022) discovered that resveratrol significantly reduced serum levels of pro-inflammatory cytokines, such as TNF-α and IL-6, by activating the SIRT1/NF-κB signaling pathway. This led to a decrease in the proportion of M1 macrophages in epididymal white adipose tissue and an increase in M2 macrophages, thus ameliorating insulin resistance. Under high glucose conditions, resveratrol can regulate macrophage polarization through the NF-κB signaling pathway. During lipopolysaccharide (LPS) stimulation, resveratrol significantly reduced nitric oxide (NO) production and the expression of pro-inflammatory cytokines IL-1 and IL-6 mRNA in LPS-stimulated macrophages. Resveratrol achieves these effects by inhibiting the NF-κB signaling pathway and activating the mTOR signaling pathway, thus promoting M2 macrophage polarization (Manríquez-Núñez et al., 2023).

However, the low oral bioavailability of resveratrol can diminish its pharmacological efficacy (de Vries et al., 2018). To overcome this challenge, resveratrol can be formulated into nanoparticles. For example, RES@PPD NPs, which are self-assembled nanoparticles from resveratrol and PPD, have been shown to inhibit the NF-κB signaling pathway, regulate host immunity, inhibit M1 macrophage polarization, and promote M2 macrophage polarization, thus treating periodontitis (Huangfu et al., 2023). These findings provide novel insights into the potential of resveratrol for treating blood sugar and dental-related inflammation, and they pave the way for improving its bioavailability. The strategic use of resveratrol, particularly through nanoparticle formulation, may offer a promising therapeutic approach for managing inflammation in metabolic and oral health disorders.

#### 3.2.2 AMPK pathway: resveratrol’s key to energy metabolism and anti-inflammatory effects

AMPK is a key regulator in cellular energy metabolism and is associated with the balance of macrophage polarization (Cheng et al., 2023). Resveratrol exerts its anti-inflammatory effects by activating the AMPK signaling pathway. Within macrophages, this mechanism promotes energy balance and metabolic regulation, thereby indirectly inhibiting inflammatory processes. It positively affects the polarization state of macrophages, helping to regulate inflammatory responses and alleviate symptoms of related diseases.

Resveratrol’s ability to modulate macrophage polarization is further supported by its effects on AMPK phosphorylation and the downregulation of p38 MAPK and JNK phosphorylation. These actions lead to a reduction in the expression of pro-inflammatory cytokines, thereby alleviating skeletal muscle inflammation in obese mice. Resveratrol prevents the accumulation of intracellular fat in skeletal muscle by suppressing the expression of toll-like receptors TLR2 and TLR4 (Shabani et al., 2020). Li et al. also revealed vascular endothelial growth factor B (VEGF-B) as a new target for resveratrol’s therapeutic action. Through the VEGF-B/AMPK/NF-κB signaling axis, resveratrol inhibits M1 macrophage polarization, which is crucial in the treatment of isoproterenol-induced myocardial injury. *In vitro* studies using RAW264.7 cells confirm that resveratrol decreases the levels of M1 markers and pro-inflammatory cytokines, while upregulating M2 markers, further contributing to the amelioration of myocardial injury (Li et al., 2020). By targeting AMPK, resveratrol promotes M2 macrophage polarization, which holds significant therapeutic potential for the treatment of periodontitis, skeletal muscle inflammation, and myocardial injury.

Tan and colleagues have conducted a groundbreaking study that demonstrates the efficacy of mesoporous silica nanoparticles (MSN) loaded with resveratrol (MSN-RSV) in the treatment of periodontitis. This novel approach diverges from previous research by highlighting a dual mechanism of action: MSN-RSV not only suppresses the NF-κB signaling pathway, which is well-known for its role in inflammation, but also activates the SIRT1/AMPK pathway, a less explored avenue in anti-inflammatory therapy (Tan et al., 2022; Wicinski et al., 2023). The synergistic effect of these two pathways not only enhances the stability of resveratrol but also extends its therapeutic window and improves bioavailability. This innovative dual-targeting strategy has been shown to inhibit M1 macrophage polarization, which is associated with pro-inflammatory responses, while promoting the alternative M2 polarization, which is anti-inflammatory and reparative. This research provides a compelling case for the use of MSN-RSV as a novel therapeutic agent with a unique dual-action mechanism, offering a promising avenue for future clinical applications in the management of these conditions.

#### 3.2.3 PI3K/Akt pathway: inducing M2 polarization or inhibiting M1 polarization for enhanced immune regulation

The PI3K/Akt pathway is a crucial regulator of macrophage responses to inflammatory signals (Yang et al., 2023). Resveratrol promotes the polarization of M2 macrophages by activating the PI3K/Akt pathway, enhancing the expression of anti-inflammatory cytokines and thereby strengthening the cell’s anti-inflammatory and immune regulatory capabilities.

Resveratrol has been the subject of extensive research due to its potential therapeutic effects. Studies have revealed that resveratrol’s activation of the PI3K/Akt signaling pathway plays a crucial role in modulating the inflammatory response. Specifically, it has been shown to suppress the secretion of pro-inflammatory factors such as TNF-α, inducible nitric oxide synthase (iNOS), and IL-1β. Concurrently, resveratrol enhances the expression of M2 macrophage markers, including Arg-1 and CD206, which are associated with anti-inflammatory and tissue repair functions (Ding et al., 2022; Hassanshahi et al., 2022). This dual action of resveratrol in regulating inflammation is particularly beneficial in the context of diabetic wound healing. By reducing the inflammatory response in wound tissue, resveratrol accelerates the healing process, which is often impeded in diabetic patients due to chronic inflammation and impaired tissue repair mechanisms. In addition to its effects on wound healing, resveratrol has also been demonstrated to have a significant impact on corneal transplant rejection. Xu et al. (2023) have reported that resveratrol can alleviate rejection by mediating the PI3K/Akt signaling pathway. This mechanism works by reducing the levels of pro-inflammatory cytokines in the corneal graft, thereby decreasing the proportion of dendritic cells in the ipsilateral cervical lymph nodes. This reduction in dendritic cells inhibits the recruitment of corneal macrophages and the polarization towards the pro-inflammatory M1 phenotype. The findings by Xu et al. are particularly noteworthy as they provide a novel approach to preventing corneal transplant rejection. By targeting the PI3K/Akt pathway, resveratrol may offer a promising strategy for immunomodulation in corneal transplantation, potentially improving graft survival rates and patient outcomes.

In conclusion, resveratrol’s ability to modulate the PI3K/Akt signaling pathway offers a multifaceted approach to managing inflammation and promoting tissue repair. Its potential applications in both diabetic wound healing and corneal transplant rejection highlight the importance of further research into this polyphenol’s therapeutic potential.

#### 3.2.4 Exploring the diverse pathways: resveratrol’s broad impact on macrophage polarization

Resveratrol, a polyphenolic compound with established anti-inflammatory properties, exerts its immunomodulatory effects through a variety of mechanisms, including the modulation of macrophage polarization. Beyond the PI3K/Akt pathway, resveratrol influences macrophage behavior by impacting metabolic pathways, the SASP pathway, and the TLR4/Myeloid differentiation primary response protein 88 (MyD88) receptor pathway. Resveratrol’s ability to modulate macrophage polarization is particularly evident in its capacity to reduce the production of pro-inflammatory cytokines such as IL-12 and NO, while simultaneously promoting the polarization towards the anti-inflammatory M2 phenotype. This effect is mediated through the alteration of macrophage metabolic pathways, which has been shown to alleviate arthritis in experimental models (Chen C. et al., 2023; Nedunchezhiyan et al., 2022; Wang and He, 2022; Zhang L. et al., 2021). Further studies have indicated that resveratrol enhances M2 polarization by increasing the expression of PPARγ, a key transcription factor associated with M2 macrophage polarization. This is accompanied by the upregulation of Arg-1, mannose receptor C type 1 (MRC-1), CCL-17, which are markers of M2 macrophages. These changes prevent obesity-related low-grade inflammation, suggesting a potential role for resveratrol in managing metabolic disorders (Ryyti et al., 2022). Yu and colleagues have demonstrated that resveratrol can modify the microenvironment by upregulating IL-10, a cytokine that promotes M2 polarization. This leads to an increase in the expression of Mrc1, Mrc2, CD163, and Arg1, while reducing the levels of iNOS, TNF-α, and MCP1. These changes are associated with improved outcomes in CCL4-induced liver fibrosis in mice, highlighting resveratrol’s potential in treating fibrotic diseases (Yu et al., 2019).

Additionally, resveratrol has been shown to reduce the expression of NO, IL-6, TNF-α, and proteins and mRNA related to the TLR4 pathway at concentrations of 2, 4, and 8 µM (Fan et al., 2021). This suggests that resveratrol promotes M2 macrophage polarization via the TLR4/MyD88 signaling pathway, thereby regulating the immune system and reducing inflammation. Under hypoxic conditions, resveratrol facilitates M2 macrophage polarization through the phosphorylation of JAK2-STAT3, which suppresses inflammatory mediators (Liu et al., 2019; Wicinski et al., 2018). This finding is significant for the treatment of myocardial infarction, where resveratrol could potentially aid in the resolution of inflammation and promote tissue repair. In summary, resveratrol’s multifaceted approach to macrophage polarization and immune modulation offers a promising avenue for the treatment of a variety of inflammatory and fibrotic diseases. Its ability to target multiple pathways and receptors underscores the potential therapeutic value of this natural compound in modulating the immune response and promoting health.

Resveratrol exhibits potent anti-inflammatory, anti-diabetic, and cardioprotective properties by modulating intracellular signaling pathways. Precisely, the intervention of resveratrol on key pathways such as NF-κB, AMPK, and PI3K/Akt plays a critical role in inducing M2 polarization or inhibiting M1 polarization, contributing to the treatment of related diseases.

## 4 Discussion and prospect

Macrophage polarization states are indeed crucial for their functions, with the M1 phenotype closely associated with anti-cancer properties. Notably, M1 macrophages can exert cytotoxic effects, which are vital for combating the growth and spread of cancer cells. Studies have shown that resveratrol can promote the polarization of macrophages towards the M1 phenotype or inhibit polarization towards the M2 phenotype, thereby exhibiting potential anti-cancer effects. This process often involves the activation of the SASP pathway or by inhibiting the activation of STAT3 and S1P-YAP signaling axes, which are key regulators of macrophage polarization. On the other hand, the M2 polarization of macrophages primarily plays an anti-inflammatory role, which is essential for preventing or alleviating inflammatory responses. M2 macrophages release anti-inflammatory cytokines and play an important role in promoting tissue repair and immune regulation. Interestingly, resveratrol can also induce the polarization of macrophages towards the M2 phenotype or inhibit polarization towards the M1 phenotype, a process that can be achieved by inhibiting the NF-κB and TLR4/MyD88 signaling pathways, which are typically associated with pro-inflammatory responses. Alternatively, this polarization can be achieved by activating the PI3K/Akt and AMPK signaling pathways, which are well-known for suppressing inflammatory responses and maintaining the body’s immune balance. Therefore, the multifaceted effects of resveratrol on macrophage polarization underscore its prospects as a therapeutic candidate for modulating immune responses.

### 4.1 Development of novel technologies to enhance resveratrol bioavailability

Resveratrol’s ability to modulate macrophage polarization offers a promising strategy for disease intervention. Determining the optimal intake of resveratrol remains a contentious issue in scientific research, with current studies proposing an experimental dose of approximately 1 g per day. However, the clinical application of resveratrol is primarily hindered by its low bioavailability. Although approximately 75% of orally administered resveratrol is absorbed in the small intestine, extensive metabolism in the gut and liver reduces its bioavailability to below 1% (Jabłońska et al., 2024). *In vitro* analyses demonstrate that resveratrol maintains relative stability during simulated gastric digestion; however, its concentration and antioxidant activity—such as DPPH radical scavenging—substantially decline during the intestinal phase. This reduction is attributed to drastic pH shifts from the stomach to the small intestine and the pH elevation induced by bile salts. Furthermore, digestive enzymes including α-amylase, pepsin, and pancreatic enzymes may hydrolyze resveratrol, compromising its structural integrity and bioactivity. Despite exhibiting some stability during *in vitro* digestion, the antioxidant efficacy of resveratrol diminishes, likely due to structural modifications and decreased bioavailability (Lee et al., 2020).

Recent investigations have highlighted the role of gut microbiota in metabolizing resveratrol precursors into resveratrol and its derivatives. Specific microorganisms, such as Slackia equolifaciens and Adlercreutzia equolifaciens, can convert resveratrol into dihydroresveratrol (DHR) (Wang et al., 2022), indicating that alterations in the digestive system significantly influence resveratrol’s bioactivity and bioavailability. Consequently, enhancing resveratrol bioavailability has become a focal point of research. Current strategies to achieve this include the development of resveratrol derivatives, co-administration with other agents, and the utilization of advanced delivery technologies (Salla et al., 2024). For example, co-administration with piperine inhibits resveratrol glucuronidation, thereby decelerating its degradation and increasing its systemic exposure—resulting in a 229% increase in area under the curve (AUC) and a 1544% increase in maximum concentration (Cmax) (Johnson et al., 2011). Additionally, nanotechnological approaches, such as solid lipid nanoparticles (SLNs) and nanostructured lipid carriers (NLCs), have been shown to effectively enhance resveratrol bioavailability (Neves et al., 2013).

### 4.2 Combined therapy of resveratrol: a new strategy to boost efficacy

To maximize its therapeutic impact and widen its application, resveratrol can be encapsulated in various pharmaceutical formulations or combined with other medications. One such innovative approach involves the integration of resveratrol into a state-of-the-art core-shell nanocomposite, QRu-PLGA-RES-DS NPs, which uses photothermal effects to stimulate M2 macrophage polarization. This strategy is particularly advantageous for managing rheumatoid arthritis and also improves the drug’s release profile (Chen et al., 2019). Additionally, a novel injectable thermosensitive hydrogel system has been engineered to include resveratrol and dexamethasone-loaded carbonate hydroxyapatite microspheres. This system is adept at promoting the repair of osteoporotic bone defects. It not only fosters the osteogenic differentiation of bone marrow mesenchymal stem cells but also mitigates the buildup of intracellular reactive oxygen species (ROS) and regulates macrophage polarization to reduce inflammation. The strategic pairing of resveratrol with dexamethasone not only amplifies the drug’s therapeutic impact but also diversifies its clinical applications (Li J. et al., 2024). This combination transcends the realm of anti-inflammatory treatments, extending to the promotion of osteogenic differentiation in bone marrow-derived stem cells, and thereby expanding the therapeutic horizons of resveratrol in clinical practice.

### 4.3 Expansion of resveratrol’s clinical applications in macrophage research

Clinical studies have demonstrated that resveratrol exhibits tissue specificity and dose dependency in cancer treatment. Notably, its metabolites accumulate in the normal tissue of prostate cancer patients (Patel et al., 2010), and higher resveratrol intake is inversely correlated with breast cancer risk (Levi et al., 2005). In obese postmenopausal women, resveratrol significantly increases sex hormone-binding globulin (SHBG) levels, which are negatively associated with breast cancer risk, without markedly affecting serum estrogen and testosterone concentrations (Chow et al., 2014). Additionally, resveratrol regulates the methylation of proteins implicated in breast cancer (Singh et al., 2015). Regarding obesity-related metabolic parameters, research findings are inconsistent. Some studies report improvements in glucose metabolism and lipid profiles (Timmers et al., 2011), whereas others find no significant anti-obesity effects (Poulsen et al., 2013). These discrepancies may stem from variations in study design, intervention duration, and resveratrol dosing. In the context of diabetes and its complications, resveratrol has shown potential therapeutic benefits by enhancing insulin sensitivity and lowering blood glucose levels (Tomé-Carneiro et al., 2013). However, outcomes vary due to factors such as racial differences and treatment duration (Fujitaka et al., 2011; Walker et al., 2019). Specifically, in diabetic patients with periodontitis, some studies report significant reductions in insulin resistance without notable changes in fasting blood glucose levels (Crandall et al., 2012; Méndez-Del Villar et al., 2014; Zare Javid et al., 2017), while others observe a decrease in fasting blood glucose (Bhatt et al., 2012; Movahed et al., 2013). These inconsistencies may be attributable to differences in study design and sample size, highlighting the need for future research to address variables such as resveratrol purity, dosage, and administration methods.

Despite significant advancements in understanding resveratrol’s pharmacological properties, clinical studies involving human participants, particularly those focusing on macrophages, remain limited. Current clinical data are constrained by short trial durations, small sample sizes, and a lack of studies explicitly evaluating health outcomes. The complexity of human physiology necessitates comprehensive investigations into the mechanisms of resveratrol action. For instance, further research is required to elucidate the molecular pathways through which resveratrol regulates blood glucose levels and ameliorates diabetes complications. Additionally, potential interactions between resveratrol and medications metabolized by enzymes such as CYP3A4 and CYP2E1 are not well understood, as many trials exclude participants on concurrent medications (Brown et al., 2024). Therefore, there is an urgent need to advance resveratrol-based therapies to ensure their efficacy and safety in clinical settings. Furthermore, studies have revealed that IL-4 induces the polarization of macrophages from the M0 to the M2 phenotype, a transition that is accompanied by notable alterations in metabolic pathways, specifically those reliant on glucose or lactate in the tricarboxylic acid cycle (Noe et al., 2021). The buildup of lactate, a defining feature of solid tumors, is intricately linked to the immunosuppressive traits of immune cells that infiltrate tumors. The potential involvement of mitochondrial metabolism of glucose or lactate in the M2 macrophage polarization induced by resveratrol remains an open mechanistic question that requires further investigation to elucidate its role in modulating the tumor microenvironment.

Significantly, a review of the literature reveals a distinct dose-dependent effect of resveratrol. At moderate concentrations, resveratrol has been shown to downregulate the expression of numerous inflammatory factors, facilitating a transition of macrophages from the pro-inflammatory M1 phenotype to the anti-inflammatory M2 phenotype. In contrast, at higher concentrations, resveratrol upregulates inflammatory factors, effectively reversing the polarization to the M1 phenotype. This concentration-dependent dichotomy highlights the importance of establishing precise pharmacological thresholds through comprehensive clinical studies. Furthermore, this intriguing phenomenon suggests new therapeutic opportunities for addressing a variety of diseases within clinical practice.

## 5 Conclusion

In this review, we have thoroughly explored the multifaceted mechanisms by which resveratrol regulates macrophage polarization through various signaling pathways, revealing its considerable potential in cancer prevention and anti-inflammatory treatments. Our findings underscore the pivotal role of resveratrol in modulating immune cell functions, thereby affirming its broad applicability and efficacy in diverse medical applications.

## Abbreviations

STAT, signal transducer and activator of transcription; JNK, c-Jun N-terminal kinase; TLR2, toll-like receptors 2; BMSC-Exos, Bone Marrow Stromal Cell-derived Exosomes; SASP, Senescence-Associated Secretory Phenotype; LPS, lipopolysaccharide; NO, nitric oxide; ROS, oxygen species; VEGF-B, vascular endothelial growth factor B; MSN, mesoporous silica nanoparticles; MSN-RSV, mesoporous silica nanoparticles loaded with resveratrol; TNF-α, tumor necrosis factor-alpha; INOS, inducible nitric oxide synthase; S1P, sphingosine-1-phosphate; AMPK, AMP-activated protein kinase; IL-10, interleukin 10; IRF-3, interferon regulatory factor 3; C/EBPβ, CCAAT/enhancer binding protein beta; PINK1, PTEN induced putative kinase 1; THP-1, Tohoku Hospital Pediatrics-1; ARG1, arginase-1; SPHK1, sphingosine kinase 1; MyD88, myeloid differentiation primary response protein 88; DHR, dihydroresveratrol; SLNs, lipid nanoparticles; NLCs, nanostructured lipid carriers; AUC, area under the curve; Cmax, maximum concentration.
